# The treatment efficacy of three-layered functional polymer materials as drug carrier for orthotopic colon cancer

**DOI:** 10.1080/10717544.2022.2122633

**Published:** 2022-09-13

**Authors:** Zhuo Liu, Dongxin Wang, Qian Cao, Jiannan Li

**Affiliations:** a Department of Gastrointestinal Colorectal & Anal Surgery, China-Japan Union Hospital of Jilin University, Changchun, China; b Department of Anesthesiology, Jilin Cancer Hospital, Changchun, China; c Department of Education, The Second Hospital of Jilin University, Changchun, China; d Department of General Surgery, The Second Hospital of Jilin University, Changchun, China

**Keywords:** Colorectal cancer, functional materials, drug release, distant metastasis

## Abstract

Colorectal cancer (CRC) is a worldwide disease posing serious threats to people’s life. Surgery and postsurgical chemotherapy are still the first choices to control the rapid progression of cancer. However, tumor recurrence and even distant metastasis are prone to occur. As a result, it is in urgent demand to find a new method to control CRC progression while inhibiting distant metastasis. On this basis, this study developed the three-layered functionalized hydrogel-fibrous membrane-hydrogel composite materials. The Chinese traditional drugs 20 (S)-ginsenoside Rg3 (Rg3) and chemotherapeutic agent 5-fluorouracil (5-Fu) were loaded in the inner hydrogel and middle fibrous membrane and could be constantly released at the same time and space. The outer hydrogel was decorated with phenylboronic acid (PA) to interact with sialic acid expressed on the CRC cell surface. The composite materials possessed biocompatibility and showed no toxicity to normal human intestinal mucosa endothelial cells HIEC. According to the results, the cell viability of CT26 could be significantly decreased *in vitro*. The three-layered functionalized materials inhibited the original tumor progression and distant tumor metastasis to the liver in an orthotopic colon cancer mouse model by increasing the caspase3 expression and inhibiting the expressions of Bcl-2, Ki-67, and VEGF. In addition, the functions of major organs were not significantly damaged. Our study provides a safe and efficacious method of this three-layered functionalized hydrogel-fibrous membrane-hydrogel composite materials for CRC treatment.

## 1. Introduction

Colorectal cancer (CRC) is a global disease and ranks third in incidence among all malignant tumors (Siegel et al., 2021). It is estimated that from 2030, 2.2 million patients will be diagnosed with CRC each year, and approximately 1.1 million patients will die of CRC (Arnold et al., 2017). Once CRC is diagnosed, surgical treatment is the first choice to control its rapid progression, after which systemic chemotherapy is often used to suppress its recurrence (Kanemitsu et al., 2021; Quenet et al., 2021). However, even if systemic chemotherapy is applied postsurgically, tumor recurrence is prone to occur in CRC (Murono et al., 2018), which may be because of the incomplete post-surgery inhibition of the activity of residual cancer cells in some cases; these cells tend to proliferate and metastasize to other sites (Power & Kemeny, 2010; Chen et al., 2021). According to statistics, about 50% of CRC patients will undergo liver metastasis, and the five-year survival rate is less than 10% (Siegel et al., 2021; Sung et al., 2021). Therefore, it is significant to develop a novel method to kill tumor tissues and capture free tumor cells to inhibit distant metastasis while decreasing side effects.

The integration of booming nanotechnology and multiple subjects provides a satisfying application prospect for the diagnosis and treatment of clinical diseases (Brede & Labhasetwar, 2013; Jin et al., 2020; Ge et al., 2021). Biodegradable polymer materials occupy an important position in nanotechnology. Polymer materials-based drug carriers, e.g., nanoparticles, hydrogel, and electrospun fibers, can load multiple drugs simultaneously, control drug release procedurally, and play different roles in many diseases (Ghafoor et al., 2018; Gagliardi et al., 2021). Electrospun nanofibers present the merits of a great drug loading rate, excellent stability, large contact area, degradability, and adjustable drug release (Feng et al., 2019). Owing to their unique structural characteristics, electrospun nanofibers provide a new strategy for various disease treatments and complication prevention. In addition, biodegradable hydrogels are also promising biomaterials owing to their hydrophilicity, biocompatibility, and non-toxicity (Parhi, 2017). Hydrogel and electrospun fibers can load agents with different hydrophilicity and hydrophobicity for local drug delivery simultaneously, which are thus often combined to provide new channels for disease treatment.

**Scheme 1.**
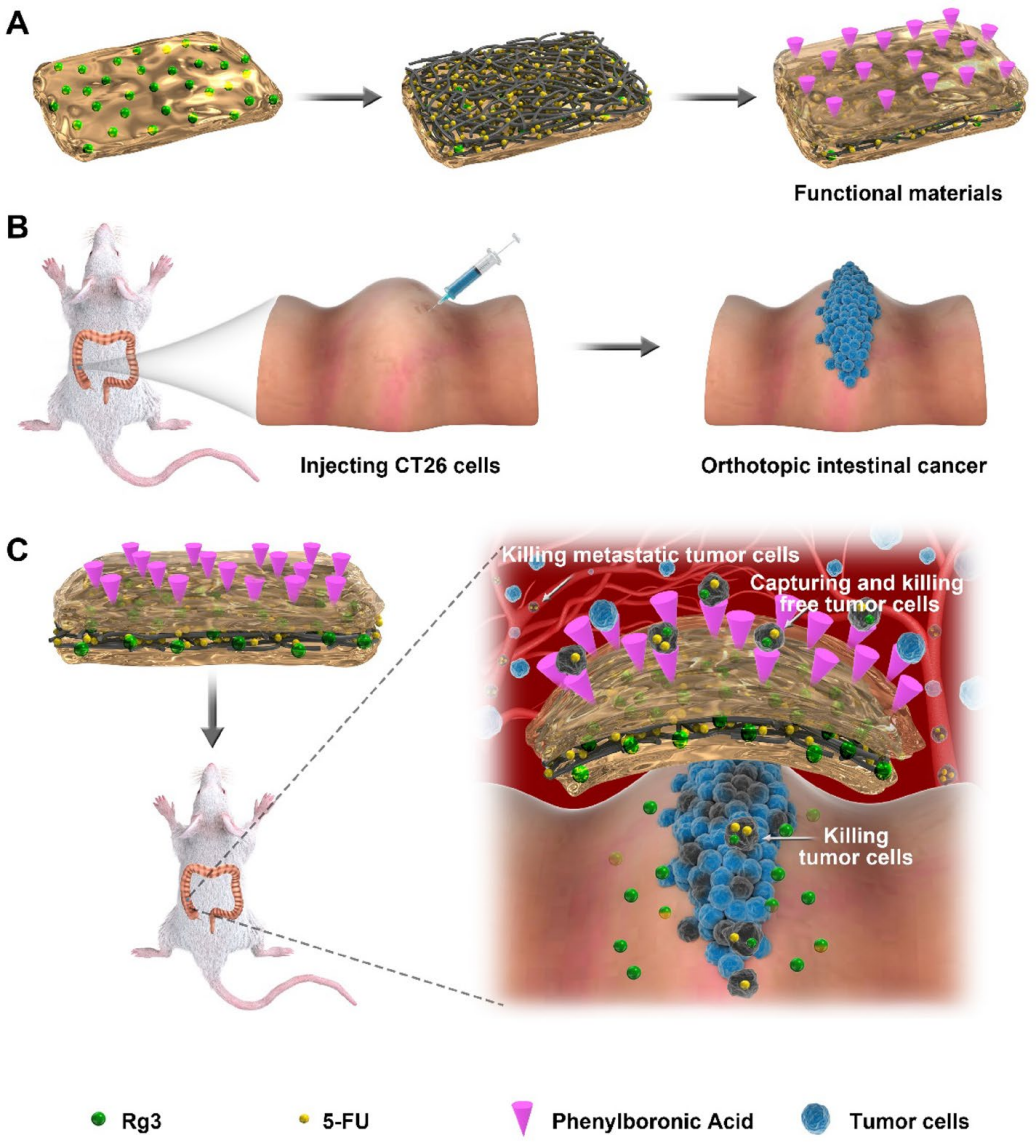
Preparation of three-layered functionalized hydrogel-fibrous membrane-hydrogel composite materials and the CRC prevention effects. (A) three-layered hydrogel-fibrous membrane-hydrogel composite materials. (B) Preparation of orthotopic colon cancer in mice. (C) Mechanisms of tumor prevention.

In this study, the three-layered functionalized hydrogel-fibrous membrane-hydrogel composite materials were developed to prevent the proliferation and distant metastasis of orthotopic colon cancer in the mouse model (Scheme 1). Based on electrospinning technology, 5-fluorouracil (5-Fu) loaded polylactic acid polyglycolic acid (PLGA) electrospun fibers were prepared. As a traditional Chinese agent, 20 (S)-ginsenoside Rg3 (Rg3) has been confirmed to inhibit tumor proliferation and metastasis by preventing angiogenesis and promoting apoptosis in many solid tumors (Zheng et al., 2017; Nakhjavani et al., 2021). Furthermore, Rg3 could enhance the synergistic anti-cancer effect of chemotherapeutic agents, such as 5-Fu and paclitaxel (Yang et al., 2012, 2019). However, Rg3 and 5-Fu present different solubility and are challenging to be integrated into one drug carrier. For this reason, in this paper, on the inner surface of the electrospun membrane, Rg3-loaded PEG–PLGA polymer hydrogel was applied. In contrast, on the outer surface, phenylboronic acid (PA) decorated PEG–PLGA polymer hydrogel was employed. Sialic acid is highly expressed on the cell surface of many cancers, including CRC (Cornelissen et al., 2019; Zhou et al., 2020). Higher expressions of sialic acid contribute to the aggressiveness and metastasis of tumor tissues (Redondo et al., 2004; Cornelissen et al., 2019). PA–sialic acid interaction can enhance the tumor-targeting effect and cause encapsulation of PA-decorated nanoparticles (Ji et al., 2016; Jeong et al., 2017). The inner hydrogel layer and middle electrospun membrane layer can achieve local release of Rg3 and 5-Fu, respectively, which synergistically inhibit tumor proliferation and angiogenesis while increasing tumor apoptosis. The free tumor cells can be captured by the PA-decorated outer hydrogel layer and subsequently killed by Rg3 and 5-Fu. In this study, the three-layered functionalized hydrogel-fibrous membrane-hydrogel composite materials were designed to prevent the progression and metastasis of orthotopic colon cancer in mice.

## 2. Materials and methods

The Material section has been provided in the Supporting file.

### 2.1. Preparation of oriented PLGA electrospun fibers

The proton nuclear magnetic resonance (^1^H NMR) of the obtained PLGA was analyzed first. The PLGA fiber membranes were prepared using an electrospinning device comprising an infusion pump, a high-voltage power supply, and a rotating grounded metallic cylindrical roller (16 cm in diameter; 22 cm in width). The specific preparation procedure was as follows. A total of 1 g PLGA was dissolved in 10 mL solution (DMF/DCM = 1/1) (10 wt.%) at about 25 °C and stirred for 2 h. Then the PLGA solution was added into a 10 mL syringe, with an advancing speed of 0.1 mm/min and high pressure of 15 kV. The electrospun membrane was collected using a roller 14 cm from the needle tip. The roller speeds were respectively preset to 1000 rpm to prepare electrospun films with orientation. To prepare 5-Fu-loaded PLGA electrospun fibers, 1 g of PLGA and 0.2 g of 5-Fu were dissolved in 10 mL of solution for further electrospun procedures. For simplicity, 5-Fu-loaded PLGA electrospun fibers were named 5-Fu-F. The 5-Fu-F morphology was analyzed by scanning electron microscopy (SEM).

### 2.2. Syntheses and characterization of PLGA-PEG-PLGA thermogel

PLGA-PEG-PLGA triblock copolymers were prepared by the ring-opening polymerization (ROP) of PEG with D,L-lactide and glycolide in the presence of stannous octoate catalyst. Generally, PEG-1000 (1 mmol), D,L-lactide (30 mmol), and glycolide (10 mmol) were added to a reaction flask. The temperature was elevated to 150 °C to melt the solid, and traces of water were removed by a high vacuum. A total of 20 uL of the toluene solution of stannous octoate (200 mg mL^−1^) was added to the mixture, which was stirred at 150 °C overnight. The crude product was dissolved in 20.0 mL of dichloromethane (DCM) and settled into 200 mL of cold diethyl ether to obtain pure PLGA-PEG-PLGA. A total of 1 g of PLGA-PEG-PLGA copolymers and 0.1 g of Rg3 were dissolved in PBS (pH 7.4) and stirred at 4 °C and changed to the hydrogel phase at 37 °C. For simplicity, the Rg3-loaded PLGA-PEG-PLGA hydrogel was named Rg3-H.

The typical ^1^H NMR and Fourier-transform infrared (FT-IR) spectra of PLGA-PEG-PLGA were investigated to analyze the structures of polypeptide thermogels.

### 2.3. Syntheses and characterization of phenylborate polypeptide thermogel

50% Phenyl borate poly (ethylene glycol)-block-poly (L-alanine) (PA-PEG-b-PLAla_28_) was synthesized via the ROP of L-alanine N-Carboxyanhydrides (L-Ala NCA) initiated by the mPEG-NH_2_ and Mal-PEG-NH_2_ (the ratio of n is 1:1). Typically, mPEG-NH_2_ (0.5 mmol) was dissolved in toluene, and azeotropic distillation was carried out for 5 h to remove the traces of water in mPEG-NH_2_. After the distillation of excess toluene under reduced pressure, N,N-dimethylformamide (DMF) was employed to re-dissolve the precipitated white solid. Mal-PEG-NH_2_ (0.5 mmol) dried by the molecular sieve and L-Ala NCA (30 mmol) were added to the above solution in N_2_ atmosphere, followed by the stirring of the mixture (25 °C, 72 h). After the reaction, the solution was poured into 500 mL of cold diethyl ether to obtain precipitation, which was dissolved in DMF. Subsequently, 4-mercaptophenylboronic acid (2 mmol) was added to the solution, which was stirred (25 °C, 12 h). Then, the solution was injected into 500 mL of cold diethyl ether, and the obtained precipitation was dissolved in DMF and further purified by dialysis against water for 3 days. Lyophilization was performed to obtain the polypeptide thermogel. For simplicity, PA-PEG-b-PLAla_28_ hydrogel was named H-PA.

The typical ^1^H NMR and FT-IR spectra of Mal-PEG-b-PLAla_28_ and PA-PEG-b-PLAla_28_ were performed.

### 2.4. Cryo-scanning electron microscopy analyses

The morphological characteristics of PA-PEG-b-PLAla_28_, PLGA-PEG-PLGA gels, and the three-layer PA-PEG-b-PLAla_28_-PLGA_8w_-PLGA-PEG-PLGA composite material were analyzed by cryogenic scanning electron microscopy (cryo-SEM), respectively. The hydrogel solution was dropped on the standard specimen holder and incubated (37 °C, 10 min). Afterward, the frozen hydrogel was broken by a scraper, sublimated to remove ice, and sprayed with platinum in the cryo-SEM sample preparation station. Finally, the samples were transferred to SEM analyses.

### 2.5. Sol-gel transition behavior

For PLGA-PEG-PLGA hydrogel, the polyester thermogels were dissolved in phosphate-buffered saline (PBS) at 15, 20, 25, and 30 wt% and stirred (72 h, 4 °C). For PA-PEG-b-PLAla_28_ hydrogel, the polypeptide thermogels were dissolved in PBS at 2, 4, and 6 wt% and stirred (48 h, 4 °C). Then, 200.0 μL of the polypeptide thermogel solution was taken and filled in a 2 mL glass vial. The phase diagrams of the polyester thermogels or the polypeptide thermogels were explored by a test tube inverting method. The temperature rose 1 °C every 5 min and was marked as the sol-gel transition point when the liquid did not flow within 30 s after inverting the vial.

### 2.6. Rheological analyses

A total of 300.0 μL of PA-PEG-b-PLAla_28_ (4 wt%) and PLGA-PEG-PLGA (25 wt%) solution was added between the parallel plates of the 25 mm diameter, and the gap was set to 0.5 mm. The sample was allowed to stand for 5 min for structure recovery and then covered with several drops of silicone oil to prevent solvent evaporation. The data of storage moduli (G′) and loss moduli (G″) were collected at a controlled strain of 1% and an angular frequency of 1.0 Hz, while the temperature was elevated from 0 to 50 °C at 0.5 °C min^−1^.

### 2.7. Preparation of Rg3-H/5-Fu-F/H-PA

5-Fu-F were cut into slides (1.5 cm × 1.5 cm, ∼30 mg), with Rg3-H (0.02 mL) and H-PA (0.02 mL) smeared on the inner face and outer face, respectively. At 37 °C, Rg3-H/5-Fu-F/H-PA was preserved for further drug release and animal study.

### 2.8. *In vitro* Rg3 and 5-Fu release

In order to evaluate the release profiles of Rg3 and 5-Fu from Rg3-H/5-Fu-F/H-PA, the *in vitro* Rg3 and 5-Fu release was investigated. In general, Rg3-H/5-Fu-F/H-PA (about 1.0 cm × 1.0 cm) was put into a glass bottle with a 20 mm diameter, followed by adding 3.0 mL of elastase solution (2.0 mg/mL, pH 7.4). The materials were incubated at 37 °C, and the solution was replaced with fresh ones daily. The collected solutions were preserved at 4 °C, and the released Rg3 and 5-Fu were analyzed by UV spectrophotometry at 254 nm and 265 nm, respectively.

The drug loading content (DLC) and drug loading efficiency (DLE) of Rg3 and 5-Fu were also assessed. DLC was calculated as the ratio of sample drug amount to drug-loaded sample amount, while DLE was expressed as the ratio of sample drug amount to the total feeding drug amount.

### 2.9. Biocompatibility analyses

PLGA-PEG-PLGA hydrogel, PEG-PLGA blank fibers, PA-PEG-b-PLAla_28_ hydrogel, and H/F/H-PA were cut into small pieces and sterilized by UV for 2 h. Normal human intestinal mucosa endothelial cells HIEC were collected and inoculated in a 24-well plate (5 × 10^4^/well). One mL of DMEM was added to each well, and cells were incubated in humidified atmosphere (5% CO_2_, 37 °C, 24 h). Different quantities of samples were added into the wells, and DMEM was replaced with fresh ones. Cells were further incubated at 5% of CO_2_ (37 °C, 72 h). Then 100 μL of MTT reagent (5 mg/mL) was added to each well and incubated for another 4 h. The supernatant and samples were removed, and then the cell pellets were dissolved in 1 mL of dimethyl sulfoxide (DMSO). The liquid in each well was removed to a 96-well plate to determine the absorbance at 490 nm using a MultiSkan plate reader (Lab systems, Helsinki, Finland). The cell viability was calculated by the ratio of the absorbance of the sample to control.

### 2.10. *In vitro* antitumor efficacy

The *in vitro* antitumor efficacy of Rg3-H/5-Fu-F/H-PA toward CT26 cells was determined by MTT method. The cells were inoculated in a 24-well plate (5 × 10^4^/well). The other procedures were kept consistent, as described in Section 2.8. The cells were treated with free Rg3 (50 μg/mL), free 5-Fu (150 μg/mL), free Rg3 + 5-Fu, and Rg3-H/5-Fu-F/H-PA (0.5 cm × 0.5 cm, ∼3.3 mg) for 72 h. The cell viability was also assessed.

### 2.11. Antitumor analyses of Rg3-H/5-Fu-F/H-PA

Balb/C mice (male, 20 g) were anesthetized via isoflurane inhalation, and then their abdominal cavities were opened through median incision. The cecum was located in the left lower abdomen and dragged out of the incision. Afterward, the colon cancer cells CT26 (1 × 10^6^) suspended in 50 μL of 0.9% saline water were injected into the subserous layer of the blood-rich area of the colon within 30 s, and a small cotton ball was used to compress the needle for 1 min. The cecum was carefully reset, and the abdominal cavity was closed. Mice were kept in plastic cages with ventilation and controlled temperature and humidity.

Seven days later, all the mice were re-anesthetized with isoflurane inhalation, and the abdomen was opened again through the same incision carefully. The colonic tumors were oval with a diameter of about 5 mm, and the mice not developing orthotopic colon tumor were excluded. For simplicity, the three-layered hydrogel-fibrous membrane-hydrogel composite materials with no drug loaded but PA decorated, with Rg3 loaded and PA decorated, with 5-Fu loaded and PA decorated, with Rg3 and 5-Fu loaded but no PA decorated, and with Rg3 and 5-Fu loaded and PA decorated were represented by H/F/H-PA, Rg3-H/F/H-PA, H/5-Fu-F/H-PA, Rg3-H/5-Fu-F/H, and Rg3-H/5-Fu-F/H-PA, respectively. We randomly divided the mice into six groups (*n* = 6): control group, H/F/H-PA group, Rg3-H/F/H-PA group, H/5-Fu-F/H-PA group, Rg3-H/5-Fu-F/H group, and Rg3-H/5-Fu-F/H-PA group. The mice in the control group received no treatment. In contrast, for mice in the other groups, about 30 mg (about 1.5 cm × 1.5 cm) of electrospun fibers (loaded with or without 5-Fu) coated with 10 mg of hydrogel (loaded with or without Rg3) on the inner side and the other 10 mg of hydrogel (decorated with or without PA) on the outer side were used. The three-layered functional materials were developed on the colonic tumor surface and were then sutured with 4-0 absorbable thread. The two kinds of hydrogel were pH sensitive and became solid phase at body temperature. After closing their abdomen, the mice were sent to cages with ventilation and adjustable temperature and humidity. The body weight of mice was recorded daily. At 14 days post-treatment, all the mice were sacrificed. The mouse blood collected from the heart before the sacrifice was centrifuged and stored. The abdomen was opened and then photographed. The intestine was resected and washed with PBS; then, it was placed in a circle and photographed. The intestinal tumors were examined, and the largest (L) and smallest (S) diameters were measured. The tumor volume (V (mm^3^)) was calculated by (L × S^2^)/2. Then the tumors were resected and weighed in each group. The intestine, together with the tumors, received H&E analysis. The diaphragm, heart, liver, spleen, lung, and kidney of the mice were resected and weighted. Histological analyses were also performed to examine the tumor invasion and metastasis as well as organ injuries. Immunohistochemical staining of caspase3, Bal-2, Ki-67, and VEGF of tumor tissues was carried out. Three different areas were randomly selected for every stained slide at high magnification (×200). A total of 500 cells were counted, and the percentage of the positively stained cells was calculated. The serum levels of CEA, ALT, AST, CEA, CK, and BUN were analyzed by ELISA methods. In addition, total RNA was isolated from the tissue samples by TRIzol (Takara, Japan) according to the manufacturer’s protocol. The mRNA detection was performed using a SYBR Green PCR Kit (Roshe, USA) with an ABI 7500 System. GAPDH expression was employed as a control for normalization. Table 1 shows the qPCR primers.

**Table 1.** The qPCR primers.

	Forward	Reverse
*Casp3*	AATGGATTATCCTGAGATGGG	GACCGAGATGTCATTCCAG
*Bcl2*	GGATGCCTTTGTGGAACTG	CAGCCAGGAGAAATCAAACAG
*Mki67*	ATACGTGAACAGGAGCCAG	CCTTGGAATCTTGAGCTTTCTC
*Vegfa*	CAGAATCATCACGAAGTGGTG	GAAGATGTCCACCAGGGTC

### 2.12. Statistical analyses

All the data were presented as mean ± standard deviation (SD). One-way ANOVA and Student *t*-test were performed. *p < .05 and **p < .01 or ***p < .001 indicated statistical significance and highly statistical significance, respectively.

## 3. Results and discussion

In this study, the three-layered hydrogel-fibrous membrane-hydrogel functionalized materials were developed. 5-Fu was loaded in the electrospun fiber layer, while Rg3 was loaded in the inner hydrogel layer. PA was used to decorate the outer hydrogel layer. The three-layered materials could well cover the orthotopic colon tumor in mice. From the materials, 5-Fu and Rg3, with different solubility, could be controllably released to synergistically inhibit tumor proliferation and angiogenesis but increase tumor apoptosis. The free tumor cells could be captured by PA-PEG-b-PLAla_28_ hydrogel in the abdominal cavity through PA–sialic acid interaction and be further killed by released 5-Fu and Rg3. Our designed materials are expected to inhibit the tumor progression and distant metastasis in the orthotopic colon cancer mouse model.

The results and analyses of ^1^H NMR and FT-IR spectra of PLGA-PEG-PLGA, ^1^H NMR of PLGA, ^1^H NMR of Mal-PEG-b-PLAla_28_ and PA-PEG-b-PLAla_28_, and FT-IR of Mal-PEG-b-PLAla_28_ and PA-PEG-b-PLAla_28_ are provided in Supporting file and shown in Figure S1.

**Figure 1.**
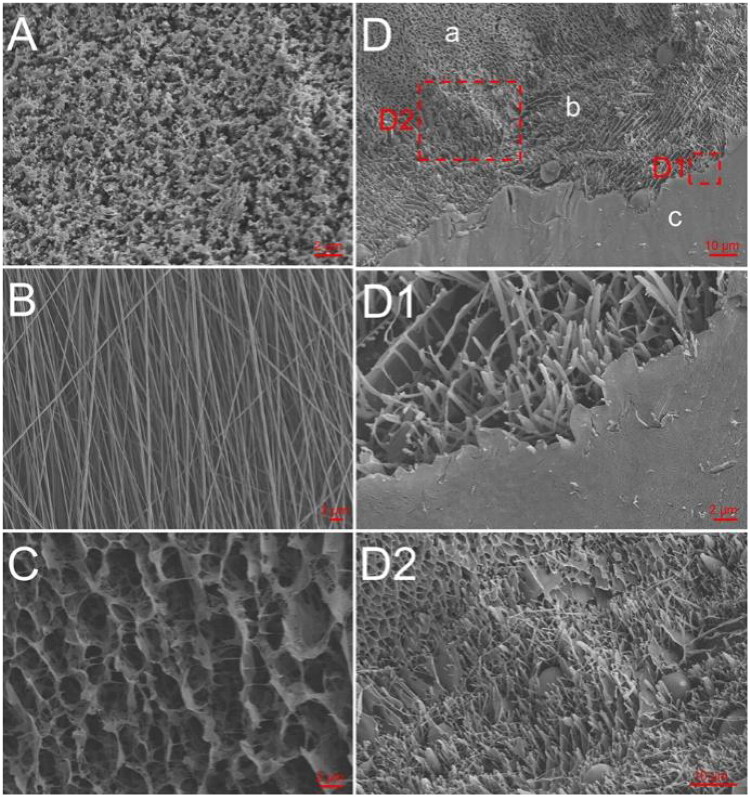
Cryo-SEM and SEM analyses. (A) Cryo-SEM of Rg3-H. (B) SEM of 5-Fu-F. (C) Cryo-SEM of H-PA. (D) Cryo-SEM of Rg3-H/5-Fu-F/H-PA; a, b, and c represent Rg3-H, 5-Fu-F, and H-PA, respectively; D1 and D2 denote the boundary interfaces of 5-Fu-F/H-PA and Rg3-H/5-Fu-F, respectively.

The morphology of Rg3-H (Figure 1A), H-PA (Figure 1C), and the Rg3-H/5-Fu-F/H-PA three-layered material (Figure 1D) were observed by cryo-SEM. The morphology of 5-Fu-F was analyzed by SEM (Figure 1B). The fibers were homogeneous with no significant drug crystallization, indicating the excellent loading of 5-Fu in the fibers. The 3D lamellar porous structure was observed in both gels. In the imaging pictures of the three-layered material, the gel-spinning-gel interface was also clearly observed (Figure 1D), proving the successful preparation of the material.

**Figure 2.**
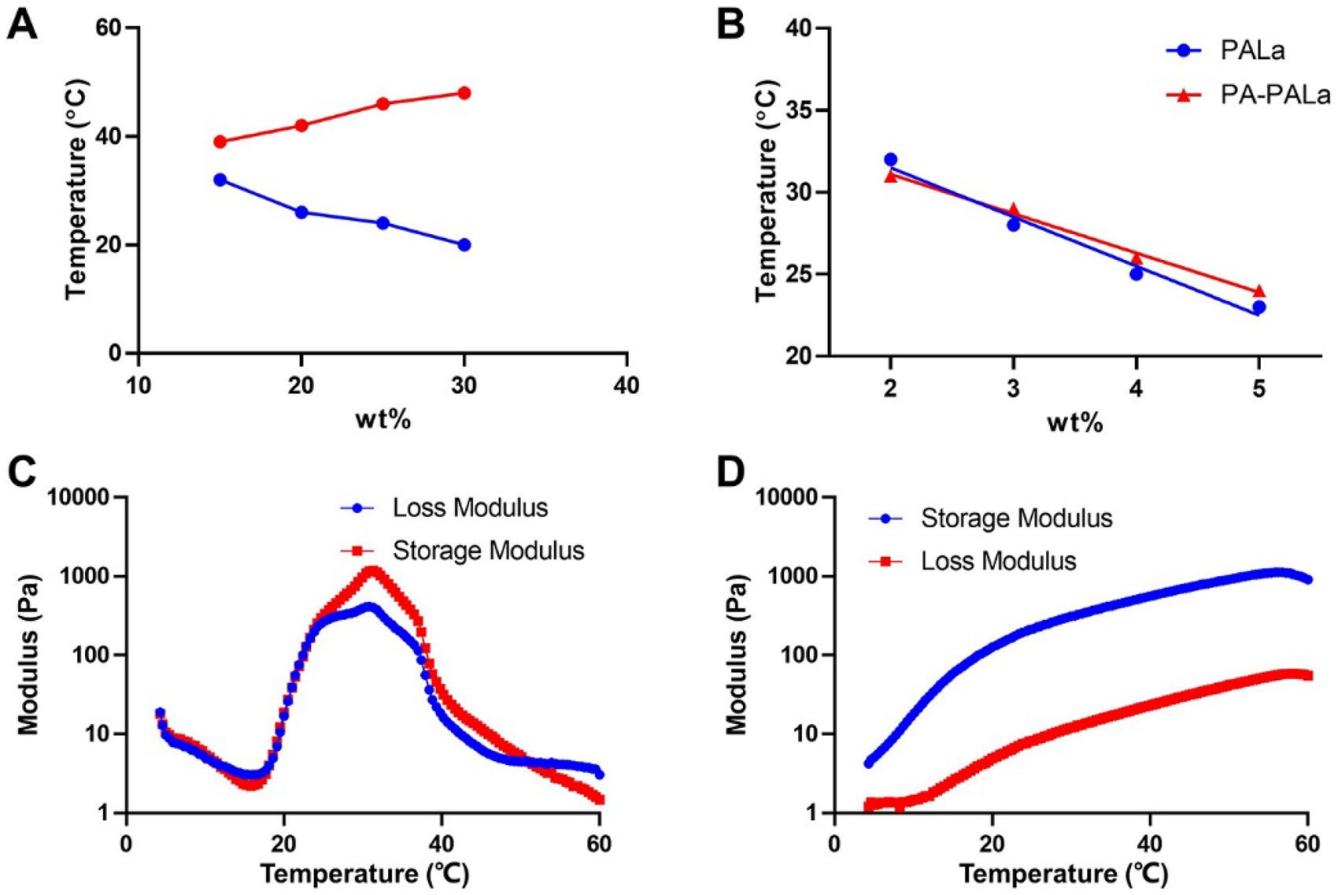
Sol-gel transition behavior (A, B) and rheological analysis (C, D) of PLGA-PEG-PLGA (A, C) and PA-PEG-b-PLAla_28_ (B, D) thermogels.

To examine the gelation behaviors of the polyester thermogel and polypeptide thermogel, the phase diagrams of PLGA-PEG-PLGA and PA-PEG-b-PLAla_28_ were tested using the tube inverting method (Figure 2A,B). The above polyester and polypeptide were dissolved in PBS (0.01 M, pH 7.4) with a concentration of 2.0–5.0 wt% and then underwent sol-gel transition with increased temperature, respectively. The appropriate concentration of PLGA-PEG-PLGA for subsequent experiments was determined to be 25 wt% (Figure 2A). The sol-gel system with 4.0 wt% polypeptides was considered optimal for application *in vivo* due to their gelation temperatures between 18 and 27 °C. It was worth noting that the modification of the PA group on the terminal of the polypeptide had a negligible effect on its gelation properties (Figure 2B). Moreover, the sol-gel system with 25.0 wt% PLGA-PEG-PLGA and 4.0 wt% PA-PEG-b-PLAla_28_ exhibited the optimum critical gelation temperatures between 20 and 25 °C for application *in vivo* (Zhang et al., 2021). In the rheological analysis of PLGA-PEG-PLGA (Figure 2C), the curves of G′ and G″ showed two crossovers during heating, demonstrating the formation of gels and precipitates, respectively. According to the rheological analysis of PA-PEG-b-PLAla_28_ (Figure 2D), the G′ value exceeded the G″ value throughout the process, proving the gel formation. Furthermore, a higher G′ value indicated a greater strength of the gel. The storage modulus of the two gels could reach ∼1,000 Pa, sufficient to meet the demand for the mechanical strength of gel application *in vivo*.

**Figure 3.**
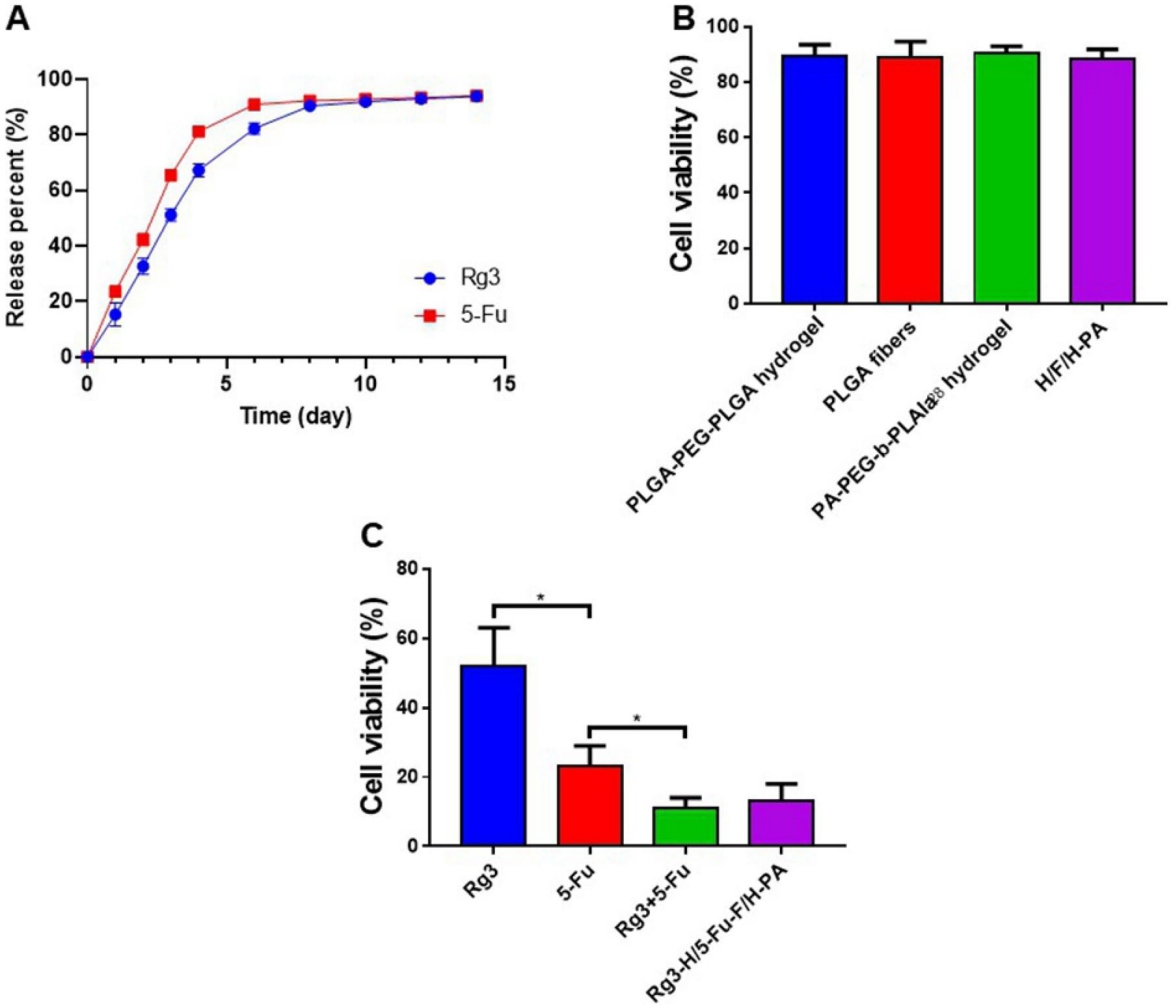
Drug release, biocompatibility, and *in vitro* antitumor analyses. (A) *In vitro* drug release of Rg3 and 5-Fu. (B) The biocompatibility analyses of PLGA-PEG-PLGA hydrogel, PEG-PLGA blank fibers, PA-PEG-b-PLAla_28_ hydrogel, and H/F/H-PA toward normal human intestinal mucosa endothelial cells HIEC. (C) The *in vitro* antitumor efficacy of Rg3-H/5-Fu-F/H-PA toward CT26 cells.

The Rg3 DLC in Rg3-H and the 5-Fu DLC in 5-Fu-F were 8.4% and 7.8%, respectively. The Rg3 DLE in Rg3-H and 5-Fu DLE in 5-Fu-F were 84.0% and 39.0%, respectively. The *in vitro* release of Rg3 and 5-Fu was investigated in pH 7.4 PBS solution because Rg3-H/5-Fu-F/H-PA was applied in the abdominal cavity (neutral environment) to cover the tumor surface. Specifically, the release profiles of Rg3 and 5-Fu lasted for about two weeks with a rapid rate in the first 4 days, a constant release in the next 3 days, and a slow rate in the remaining days (Figure 3A). The rapid release of Rg3 and 5-Fu was attributed to the porous structure and large surface area of PLGA-PEG-PLGA hydrogel and PLGA electrospun membrane and the drug distribution near the surface of hydrogel and nanofibers (Jacob et al., 2021; Luraghi et al., 2021). In our study, Rg3 and 5-Fu were loaded in different layers of Rg3-H/5-Fu-F/H-PA, enabling the two drugs with different solubility to be continuously released at the same time and space, beneficial for tumor inhibition effect. The biocompatibility of the nanomaterials was important for further application. After 72 h incubation, the cell viabilities of normal human intestinal mucosa endothelial cells HIEC were above 90%, and no statistical difference was found in cell viability among these four groups (Figure 3B). This suggested that PLGA-PEG-PLGA hydrogel, PEG-PLGA blank fibers, PA-PEG-b-PLAla_28_ hydrogel, and H/F/H-PA were biocompatible and safe for *in vivo* application. The *in vitro* antitumor efficacy of Rg3-H/5-Fu-F/H-PA toward CT26 cells was identified. After 72 h incubation, CT26 cells in Rg3-H/5-Fu/H-PA and Rg3 + 5-Fu treated groups represented a significantly suppressed cell viability, but there was no difference between the two groups (Figure 3C). The cell viability in Rg3 + 5-Fu treated group was lower than that of both the 5-Fu and Rg3 treated groups. The *in vitro* antitumor study demonstrated that the functionalized three-layered Rg3-H/5-Fu/H-PA material could decrease the cell viability of CT26 cells, which is vital for the *in vivo* antitumor application of the material.

**Figure 4.**
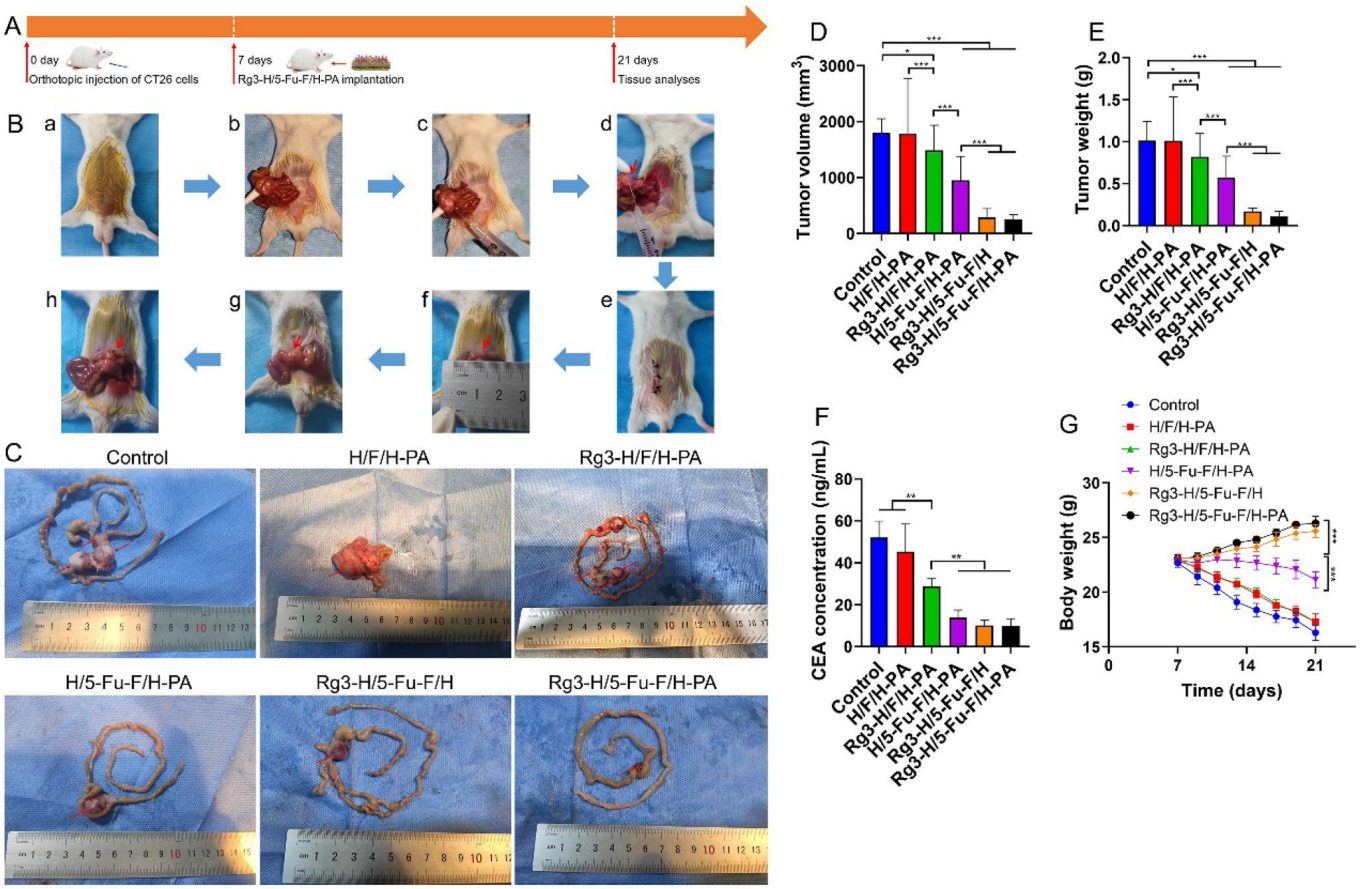
Antitumor analyses of Rg3-H/5-Fu-F/H-PA. (A) Experimental schedule. (B) The detailed establishment procedure of the orthotopic colon cancer mouse model and Rg3-H/5-Fu-F/H-PA implantation. a. Abdominal disinfection; b. exposure of the colon; c. injection of CT26 cells into the subserous layer of the blood-rich area of the colon; d. tumor cells were injected, and the red arrow indicated the tumor cell solution within the subserous layer of the colon; e. the closure of the abdominal incision; f. the tumor (red arrow) formed at 7 days later; g. Rg3-H/5-Fu-F/H-PA (red arrow) was covered on the tumor surface; h. Rg3-H/5-Fu-F/H-PA was sutured with nearby colon serosa, and the red arrow indicated the suture. (C) Photographs of the whole intestine and the intestinal tumors. Red arrows indicated the intestinal tumors. (D) Tumor volume. (E) Tumor weight. (F) Blood CEA concentration. (G) Body weight changes. For each group in D, E, F, and G, *n* = 6.

To evaluate the antitumor efficacy of Rg3-H/5-Fu-F/H-PA, the orthotopic colon cancer mouse model was developed (Figure 4A). Seven days later, the tumors formed, and mice with tumor diameters of about 5 mm were selected. The tumor surface was treated with Rg3-H/5-Fu-F/H-PA, which lasted for another 14 days. The detailed procedures of orthotopic colon cancer mouse model establishment and Rg3-H/5-Fu-F/H-PA implantation are provided in Figure 4(B). At 14 days post-treatment, the tumors were large and occupied nearly the whole abdomen in the control and H/F/H-PA groups. In the other four groups, when the abdomen was open, the intestinal tumors cannot be exposed clearly because of the coverage of normal intestine. In addition, in four mice in the control group and three mice in the H/F/H-PA group, the tumor tissues invaded the diaphragm, and thus sharp separation should be performed to dissect the tumors. The photographs of the resected large intestine and small intestine were obtained (Figure 4C). In the Rg3-H/F/H-PA and H/5-Fu/H-PA groups, multiple intestinal tumors could be found in certain mice, while independent tumors were observed in others. The tumors in these two groups did not occupy too much space; that is, the tumors did not invade the diaphragm or abdomen of the mice. In the Rg3-H/5-Fu-F/H and Rg3-H/5-Fu-F/H-PA groups, the tumor tissues were almost independent in each mouse, and the tumors were significantly inhibited. For a statistical comparison between the tumors, the tumor volume (Figure 4D) was calculated, and the tumor weight (Figure 4E) was recorded. No statistical difference existed in tumor volume and tumor weight between the control and H/F/H-PA groups. Compared with the control group, Rg3-H/F/H-PA, H/5-Fu-F/H-PA, Rg3-H/5-Fu-F/H, and Rg3-H/5-Fu-F/H-PA groups showed less tumor volume and tumor weight. Both the tumor volume and tumor weight were lower in the Rg3-H/F/H-PA group than in the H/F/H-PA group (***p < .001), demonstrating the antitumor effect of the released Rg3 from the inner hydrogel. The mice in the H/5-Fu/H-PA group showed a better tumor prevention effect than those in the Rg3-H/F/H-PA group because a significant difference existed in tumor volume and tumor weight between the two groups (***p < .001). As the first-line chemotherapeutic agent against CRC, 5-Fu was successfully loaded in the middle electrospun membrane and continuously released owing to its antitumor efficacy. The tumor volume and tumor weight were the smallest in Rg3-H/5-Fu-F/H and Rg3-H/5-Fu-F/H-PA groups, but no difference was found between these two groups. Because 5-Fu and Rg3 possess different solubility and can hardly be loaded on one platform, they were loaded in the inner hydrogel and the middle electrospun membrane, respectively, for the benefit of their release at the same time and space. It has been reported that Rg3 can prevent angiogenesis and the progression of multiple solid tumors and increase apoptosis (Zheng et al., 2017; Nakhjavani et al., 2021). Yang demonstrated a synergistic anticancer effect of 5-Fu plus Rg3, and the co-treatment worked more effectively than either drug alone in decreasing the viability of HCT cell lines (Yang et al., 2019). Similarly, in our study, the combination of these two agents also showed a better tumor prevention effect than either one, supporting the synergistic anticancer effect of 5-Fu and Rg3. The outer hydrogel layer was decorated by PA and was designed to capture free tumor cells and inhibit further tumor metastasis. However, the PA-decorated outer hydrogel layer failed to result in the downregulation of tumor volume and tumor weight in our study. CEA is a kind of immunoglobulin cell adhesion molecule, which is elevated in the serum in multiple cancers, especially in CRC (Spindler et al., 2017). The blood CEA concentration is shown in Figure 4(F). The CEA concentrations were the poorest in H/5-Fu/H-PA, Rg3-H/5-Fu-F/H, and Rg3-H/5-Fu-F/H-PA groups, which presented no difference. The Rg3-H/F/H-PA group showed a lower CEA concentration than the control and H/F/H-PA groups (**p < .01). The body weight of each mouse was also recorded (Figure 4G). As can be seen, due to the tumor cachexia, the body weight in the control, H/F/H-PA, Rg3-H/F/H-PA, and H/5-Fu-F/H-PA groups dropped significantly. At 14 days post-treatment, the body weight of mice was significantly higher in the H/5-Fu-F/H-PA group than in the Rg3-H/F/H-PA group. In addition, the body weight of mice in the Rg3-H/5-Fu-F/H and Rg3-H/5-Fu-F/H-PA groups was elevated continuously and no difference appeared at 14 days post treatment; the enhanced body weight in the two groups demonstrated a better general body condition than mice in other groups. In the other four groups, the decreased body weight may be explained by the tumor cachexia supported by the large tumor volume and high tumor weight.

**Figure 5.**
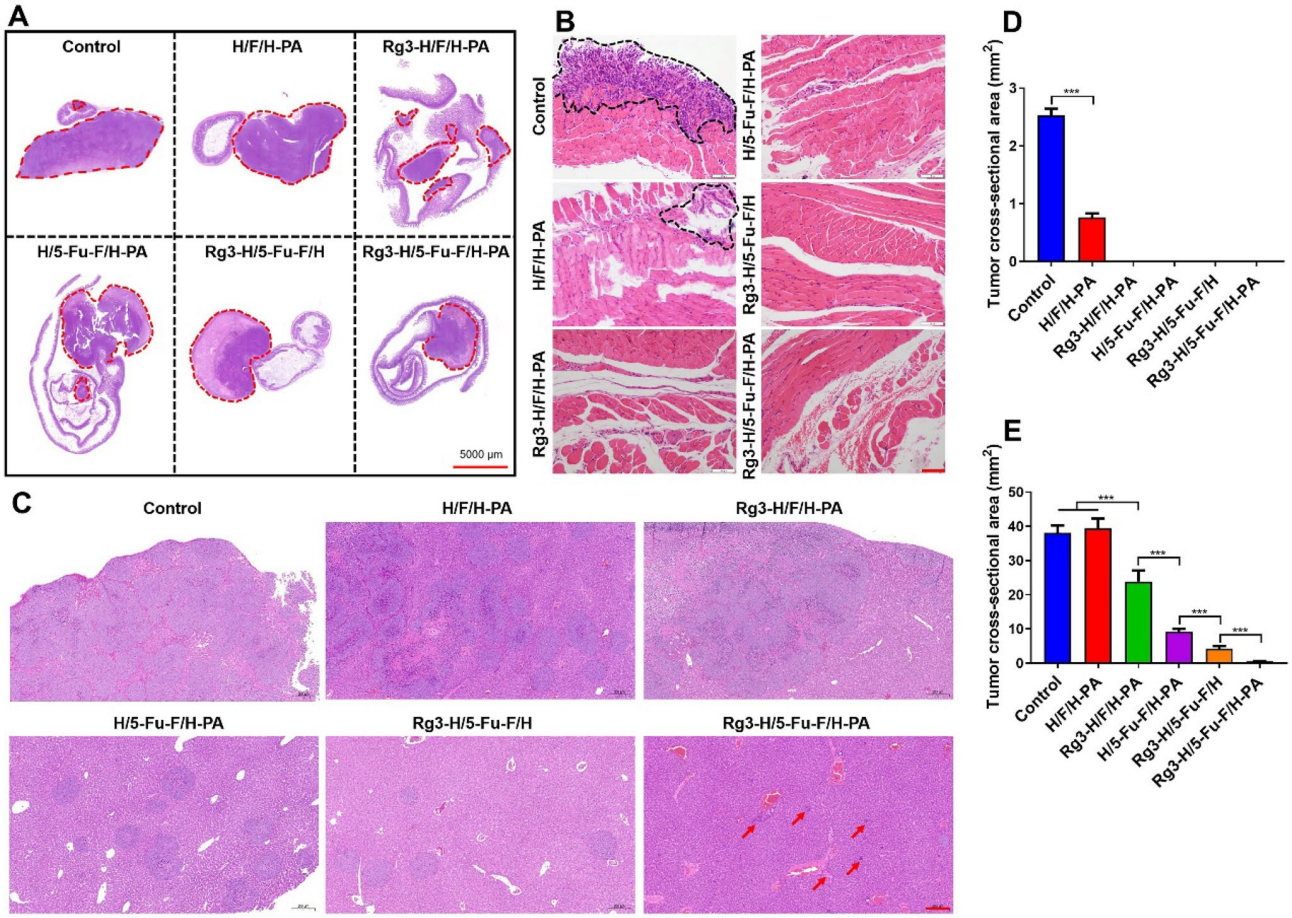
Histopathological analyses of the antitumor efficacy of Rg3-H/5-Fu-F/H-PA. (A) H&E staining of the intestine and tumor tissues. Red circles indicated the tumor tissues. (B) H&E staining of the diaphragm. Black circles indicated the invaded tumor tissues. Scale bar = 100 μm. (C) H&E staining of liver tissues. Scale bar = 200 μm. (D) Cross-sectional tumor area in the diaphragm. (E) Cross-sectional tumor area in liver. For control and H/F/H-PA groups in D, *n* = 3. For each group in E, *n* = 6.

In order to further evaluate the anticancer effect of Rg3-H/5-Fu-F/H-PA, histopathological analyses were performed. H&E staining of the intestinal tumors and adjacent intestine was carried out (Figure 5A). In all groups, the tumor tissue-normal intestine margins were sharp. Large apoptosis and necrosis areas were observed in tumor tissues in H/5-Fu-F/H-PA, Rg3-H/5-Fu-F/H, and Rg3-H/5-Fu-F/H-PA groups. The cross-sectional tumor area for the original intestinal tumors was not analyzed because the overlarge tumor volumes in control and H/F/H-PA groups prohibited all the tumor tissues from being embedded within the wax block. The tumor invaded the diaphragm in the control and H/F/H-PA groups, which was confirmed by the H&E staining of the diaphragm (Figure 5B). But in the other four groups, the tumors did not invade the diaphragm and no signs of tumor metastasis to the diaphragm was found. The cross-sectional tumor area of the invaded tumor tissues in the diaphragm was analyzed by Adobe Photoshop CS6 (Adobe, California, USA), which was larger in the control group than that in the H/F/H-PA group (***p < .001) (Figure 5D). The liver is the first and major organ for the metastasis of colon tumor cells (Yoshida et al., 2012), and the H&E staining of liver tissues was performed (Figure 5C). A large area of metastatic tumor tissues could be observed in the liver of mice in control, H/F/H-PA, and Rg3-H/F/H-PA groups. In contrast, scattered and small areas of metastatic tumor tissues were observed in the liver in H/5-Fu-F/H-PA and Rg3-H/5-Fu-F/H groups. Only a small number of metastatic tumor cells were found in the Rg3-H/5-Fu-F/H-PA group. The cross-sectional area of metastatic tumor tissues in the liver is shown in Figure 5(E). The Rg3-H/5-Fu-F/H-PA group represented the least tumor cross-sectional area in the liver among all the groups. In particular, a significant difference existed between Rg3-H/5-Fu-F/H and Rg3-H/5-Fu-F/H-PA groups (***p < .001). This indicated the optimal tumor metastasis inhibition effect of Rg3-H/5-Fu-F/H-PA in our study. The reason was that the PA-decorated outer layer hydrogel could capture free tumor cells as a result of PA–sialic acid interaction. The captured tumor cells could be further killed by the released 5-Fu and Rg3. In addition, the cross-sectional tumor area was smaller in the Rg3-H/5-Fu-F/H groups than that in H/5-Fu-F/H-PA and Rg3-H/F/H-PA groups (***p < .001). The combination of 5-Fu and Rg3 could perform a synergistic anticancer effect than either of the two agents, thus decreasing the metastasis of tumor cells. Taken together, the 5-Fu- and Rg3-loaded, PA-decorated, three-layered functionalized nanomaterials possessed satisfactory effects on tumor inhibition and distant metastasis prevention.

**Figure 6.**
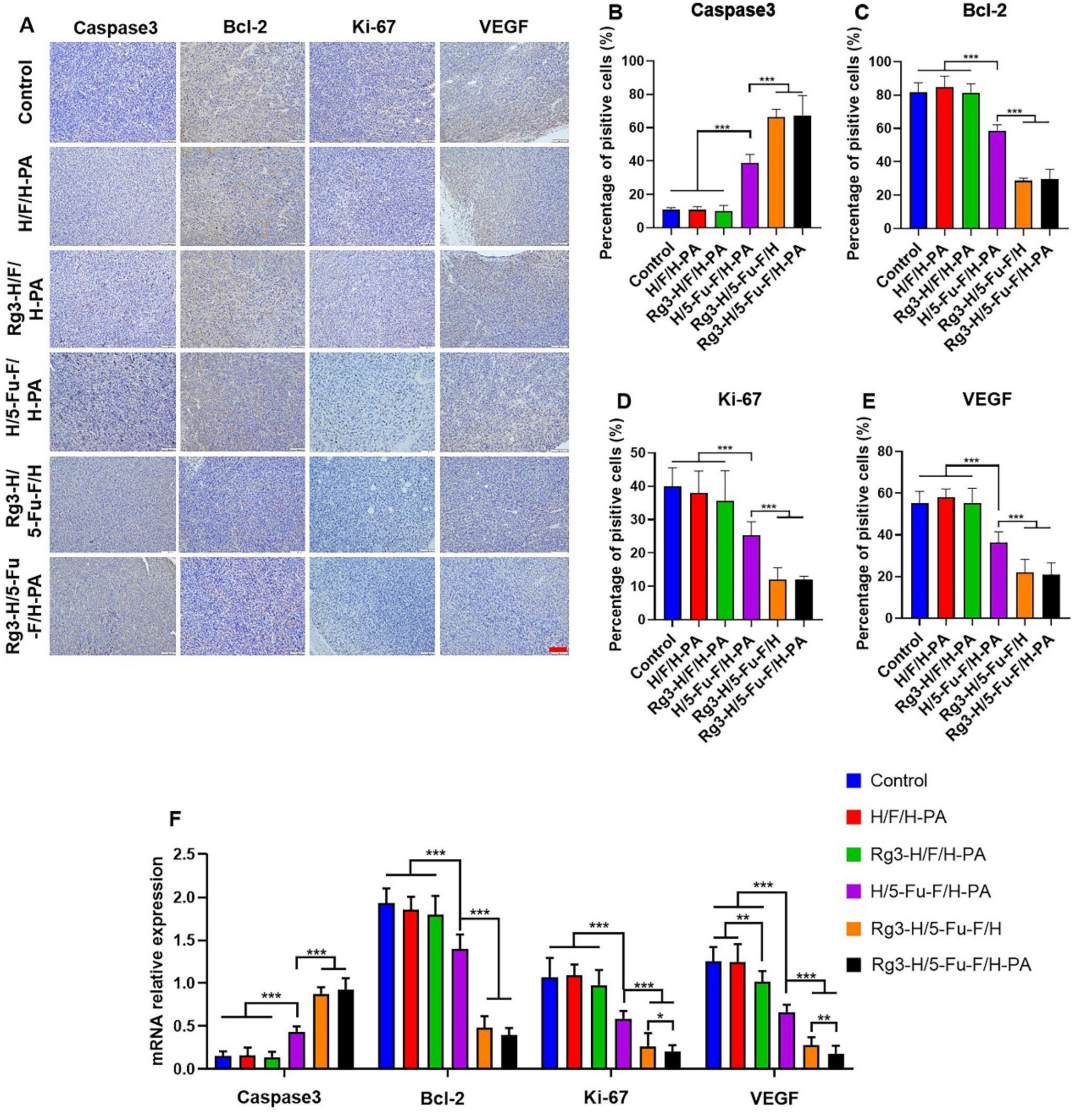
Immunohistochemical analyses of the antitumor mechanisms of Rg3-H/5-Fu-F/H-PA. (A) Immunohistochemical analyses of caspase3, Bcl-2, Ki-67, and VEGF. Scale bar = 100 μm. Percentage of positively stained cells of (B) caspase3, (C) Bcl-2, (D) Ki-67, and (E) VEGF. (F) RT-PCR analysis. For each group in B, C, D, E, and F, *n* = 6.

Immunohistochemical staining was conducted to further demonstrate the anticancer effects of Rg3-H/5-Fu-F/H-PA. The expression levels of apoptosis-related protein caspase3 and antiapoptosis-related protein Bcl-2 were analyzed. High expression of caspase3 and low expression of Bcl-2 can indicate mass apoptosis of tumor cells. Only minimal cells were stained brown for caspase3 in the control, H/F/H-PA and Rg3-H/F/H-PA groups (Figure 6A), and there was no statistical difference in the percentage of positively stained cells among the three groups (Figure 6B). More positive caspase3 stained cells were observed in the H/5-Fu-F/H-PA group, in which the percentage of positively stained cells was higher than that in the Rg3-H/F/H-PA group (***p < .001) (Figure 6B). The percentage of positive caspase3 stained cells was the greatest in Rg3-H/5-Fu-F/H and Rg3-H/5-Fu-F/H-PA groups, between which no difference existed (Figure 6B). The Bcl-2 expression levels were the poorest in Rg3-H/5-Fu-F/H and Rg3-H/5-Fu-F/H-PA groups, with no difference found (Figure 6A,C). The percentage of positive Bcl-2 stained cells was lower in the H/5-Fu-F/H-PA group than that in the control, H/F/H-PA, and Rg3-H/F/H-PA groups (***p < .001) (Figure 6C). These results demonstrate that Rg3-H/5-Fu-F/H and Rg3-H/5-Fu-F/H-PA can induce the tumor apoptosis by increasing caspase3 levels and decreasing Bcl-2 levels. Ki-67 is a kind of protein associated with cell proliferation (Niotis et al., 2018). The expression levels of Ki-67 were the lowest in Rg3-H/5-Fu-F/H and Rg3-H/5-Fu-F/H-PA groups, which showed no difference (Figure 6A,D). Furthermore, no difference was found in the percentage of positive Ki-67 stained cells in the control, H/F/H-PA and Rg3-H/F/H-PA groups (Figure 6D). The Ki-67 expression level was lower in the H/5-Fu-F/H-PA group than in the Rg3-H/F/H-PA group (***p < .001) (Figure 6D). VEGF is a kind of protein crucial for tumor angiogenesis (Nalli et al., 2021). The VEGF expression level was the lowest in Rg3-H/5-Fu-F/H and Rg3-H/5-Fu-F/H-PA groups (Figure 6A,E). The percentage of positive VEGF stained cells was poorer in the H/5-Fu-F/H-PA group than in the control, H/F/H-PA, and Rg3-H/F/H-PA groups (***p < .001). The RT-PCR results are similar with that of immunohistochemical analysis (Figure 6F). In detail, Rg3-H/5-Fu-F/H and Rg3-H/5-Fu-F/H-PA groups showed the most caspase3 and the least Bcl-2 levels, compared with all the other groups. As for Ki-67 and VEGF expression, Rg3-H/5-Fu-F/H-PA showed the most inhibition effect and there was significant difference between Rg3-H/5-Fu-F/H and Rg3-H/5-Fu-F/H-PA groups. These results indicated that Rg3-H/5-Fu-F/H-PA inhibited the progression of orthotopic colon cancer in mice through activating tumor apoptosis as well as inhibiting tumor cell proliferation and tumor angiogenesis. Compared with Rg3-H/F/H-PA and H/5-Fu-F/H-PA, Rg3-H/5-Fu-F/H-PA presented greater promotion of tumor apoptosis and inhibition of tumor proliferation and angiogenesis. This demonstrated the successful application of the combination of 5-Fu and Rg3 in our functionalized materials to render a synergistic anticancer effect.

**Figure 7.**
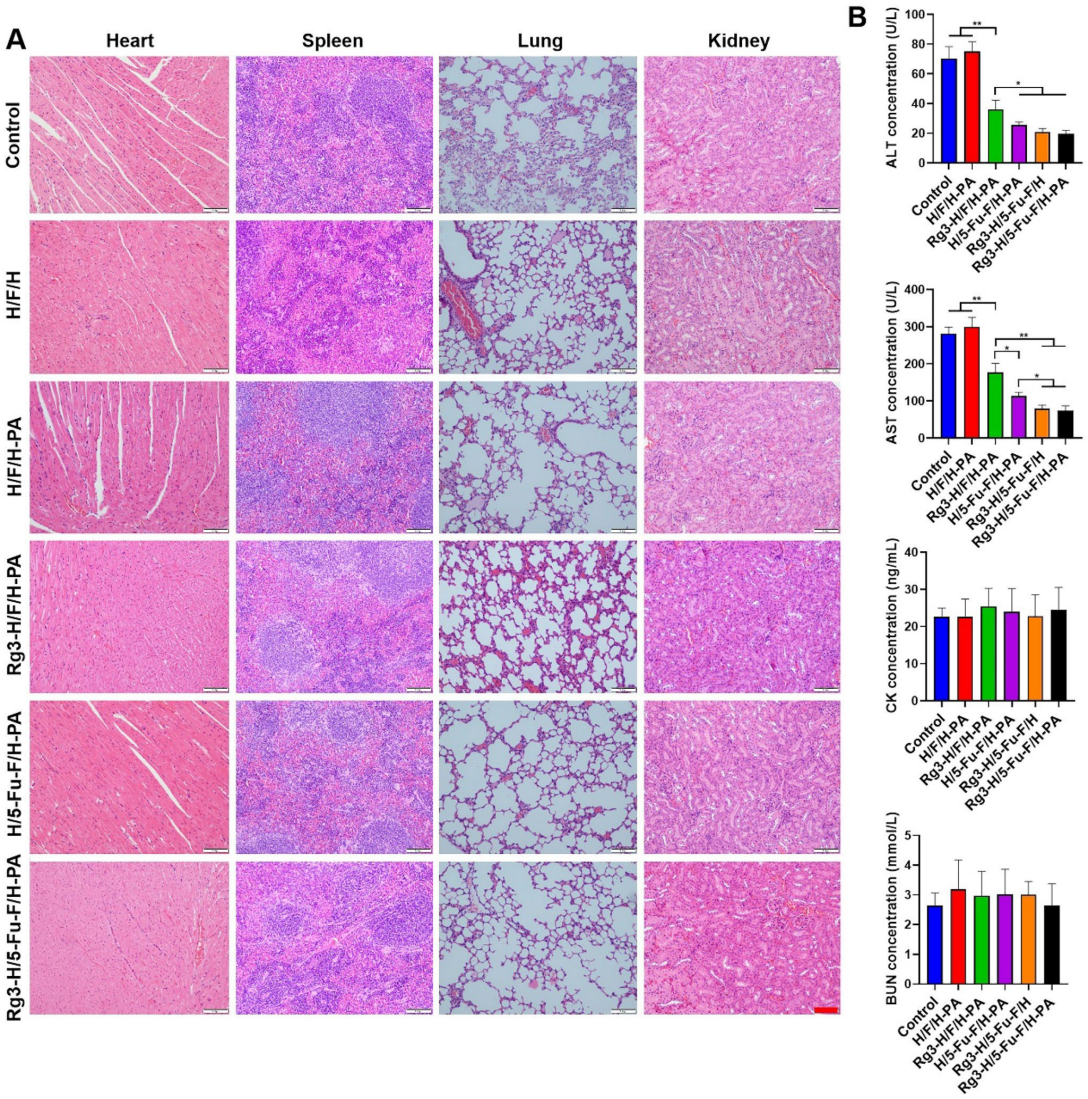
Application safety analyses of Rg3-H/5-Fu-F/H-PA *in vivo*. (A) H&E staining of major organs. Scale bar = 100 μm. (B) Serum levels of ALT, AST, CK, and BUN. For each group in B, *n* = 6.

In order to evaluate the application safety of Rg3-H/5-Fu-F/H-PA *in vivo*, histopathological analysis of the major organs was performed. H&E evaluation of the heart, spleen, lung, and kidney did not reveal any injuries or tumor cells (Figure 7A). As for the blood biochemical analysis, the ALT and AST levels were significantly higher in control and H/F/H-PA groups than all the other groups because of tumor metastasis to liver tissues (Figure 7B). Rg3-H/F/H-PA group showed higher levels of ALT and AST than H/5-Fu-F/H-PA, Rg3-H/5-Fu-F/H, and Rg3-H/5-Fu-F/H-PA groups. No difference was observed among H/5-Fu-F/H-PA, Rg3-H/5-Fu-F/H, and Rg3-H/5-Fu-F/H-PA groups in terms of the ALT levels or between Rg3-H/5-Fu-F/H and Rg3-H/5-Fu-F/H-PA groups in terms of the AST levels. This indicated that the liver function of mice was seriously damaged because of tumor metastasis, especially the mice in control, H/F/H-PA, and Rg3-H/F/H-PA groups. However, compared with other groups, less tumor metastasis occurred in the liver of mice in Rg3-H/5-Fu-F/H and Rg3-H/5-Fu-F/H-PA groups, which presented better liver function. No statistical difference existed in CK and BUN levels among all the groups, indicating the undamaged heart and kidney. Our results suggested that Rg3-H/5-Fu-F/H-PA could be safely applied *in vivo* without inducing obvious injuries to major organs.

There are some limitations of this study. (1) The error bars of H/F/H-PA groups in Figure 4(D,E) are a bit high, which may decrease the homogeneity. However, this did not influence the final demonstration of the tumor inhibition effect of Rg3-H/5-Fu-F/H-PA. (2) In Figure 5(C), the tumor sites are scattered in H/F/H-PA group and the whole liver section cannot be photographed in one image at a fixed magnification. However, for the analysis of tumor cross-sectional area, all the metastatic tumor sites should be included. As a result, it seems that there is some difference in the metastatic tumor area between the control and H/F/H-PA groups in Figure 5(C) which is different from the result in Figure 5(E). Actually, the metastatic tumor cross-sectional areas between these two groups are the same. (3) The pharmacokinetics analysis was not performed in this study. In our system, the agents released from Rg3-H/5-Fu-F/H-PA act directly on the tumor site, which is equivalent to *in situ* drug delivery. Different from conventional small-molecule drugs and nanomaterials, which allow drugs to reach the lesion site through systemic circulation, our study pays more attention on improving drug utilization and treatment efficacy by delivering drugs *in situ.*

## 4. Conclusions

In this study, the three-layered functionalized hydrogel-fibrous membrane-hydrogel composite materials were developed to inhibit tumor progression and distant metastasis in the orthotopic colon cancer mouse model. Moreover, 5-Fu and Rg3 could be released continuously from Rg3-H/5-Fu-F/H-PA to achieve a synergistic anticancer effect by activating tumor apoptosis as well as inhibiting tumor cell proliferation and tumor angiogenesis. Rg3-H/5-Fu-F/H-PA could lessen distant tumor metastasis because the PA-decorated outer hydrogel could capture free tumor cells based on PA–sialic acid interaction. All the results indicated the efficacy of the materials for colon cancer treatment.

## Supplementary Material

Supplemental Material
