# Supplementary material for: The treatment efficacy of three-layered functional polymer materials as drug carrier for orthotopic colon cancer
Source: Drug Deliv. 2022 Sep 13;29(1):2971–83. doi: 10.1080/10717544.2022.2122633 (PMC9487963; doi:10.1080/10717544.2022.2122633)
Supplement: Supplemental Material [file IDRD_A_2122633_SM6440.docx]

The treatment efficacy of three-layered functional polymer materials as drug carrier for orthotopic colon cancer

Zhuo Liu^1^, Dongxin Wang^2^, Qian Cao^3^, Jiannan Li^4,^*

^1^Department of Gastrointestinal Colorectal & Anal Surgery, China-Japan Union Hospital of Jilin University, Changchun130033, China

^2^Department of Anesthesiology, Jilin Cancer Hospital, Changchun 130031, China

^3^Department of Education, Jilin University Second Hospital, Changchun 130041, China

^4^Department of General Surgery, Jilin University Second Hospital, Changchun 130041, China

*Corresponding author

*Email address:* [jnli@jlu.edu.cn](mailto:jnli@jlu.edu.cn), jnli@ciac.ac.cn (Jiannan Li)

Materials

Methoxy poly(ethylene glycol) (mPEG; number-average molecular weight (Mn) = 1,000 g mol^−1^), terminal aminated poly(ethylene glycol) (mPEG-NH_2_; number-average molecular weight (Mn) = 2,000 g mol^−1^), and terminal aminated poly(ethylene glycol) maleimide (Mal-PEG-NH_2_; number-average molecular weight (Mn) = 2,000 g mol^−1^) were obtained from Sigma-Aldrich (Shanghai, P. R. China). L-Alanine N-carboxyanhydride (L-Ala NCA) was purchased from Chengdu Enlai Biological Technology Co., Ltd. (Chengdu, P. R. China). Triphosgene was obtained from Shanghai Duodian Chemical Co., Ltd. (Shanghai, P. R. China). Lactide and glycolide were purchased from Aladdin (Shanghai, P. R. China). Stannous octoate were purchased from Sigma Aldrich (Shanghai, P. R. China). All the other chemicals were purchased from Beijing Chemical Industry Group Co., Ltd. (P. R. China). Poly(lactic-co-glycolic acid) (PLGA: LA/GA=3/1; number-average molecular weight (Mn) = 80,000 g mol^−1^) was obtained from Jinan Daigang Biomaterial Co., Ltd. (Shandong, P. R. China). HIEC and CT26 cell lines were purchased from Cybertron Biotechnology Company (Shanghai, China). CEA, ALT, AST, CEA, and BUN ELISA kits were bought from Senbeijia Biotechnology Company (Wuhan, China). The primary antibodies of caspase-3, Bcl-2, Ki-67, and vascular endothlial growth factor (VEGF) were purchased from Beijing Bioss Technology Company (Beijing, China).

Results and Discussion

The ^1^H NMR and FT-IR spectra of PLGA-PEG-PLGA were analyzed to confirm the chemical structures of polypeptide thermogels. As shown in Figure S1A, the characteristic peaks at 1.60 and 5.22 ppm were assigned to the protons of methyl group and methine group in PLA. The peaks at 4.83 ppm attributed to the protons of methylene group in PGA. The peaks at 4.33 ppm attributed to the protons of methylene group attached to ester bond in mPEG. The peaks at 3.66 ppm were assigned to the protons of mPEG backbone. The specific molecular formula of the thermogel is PLGA1600-PEG1000-PLGA1600 (LA/GA=33/12), which was calculated by integrating the peak areas. Furthermore, the FT-IR spectra were collected to verify the chemical structures of the thermogels further. As shown in Figure S1B, the signals at 1760.2 cm^−1^ and 1188.0 cm^−1^ attributed to stretching vibration of the ester bond (νC=O) and (νC(O)-O) of backbone further validated the chemical structures of polyester. The signals at 1093.5 cm^−1^ attributed to stretching vibration of the ether bond (νO-C-O) of PEG. This confirmed that PLGA-PEG-PLGA thermogel was successfully synthesized. The ^1^H NMR spectrum was analyzed to confirm the chemical structures of PLGA_8w_. As shown in Figure S1C, the characteristic peaks at 1.6 and 5.2 ppm were assigned to the protons of methyl group (−C(O)CH(C*H*_3_)O−, c) and methine group (−C(O)C*H*(CH_3_)O−, a) in PLA. The peaks at 4.8 ppm were assigned to the protons (−C(O)C*H*_2_O−, b) of methylene group in PGA. The ^1^H NMR and FT-IR spectra of Mal-PEG-b-PLAla_28_ and PA-PEG-b-PLAla_28_ were analyzed to confirm the chemical structures of polypeptide thermogels. As shown in Figure S1D, the characteristic peaks at 1.36 and 4.47 ppm were assigned to the protons of methyl group in alanine and methine in the backbone of PAla. The peaks at 3.41 ppm and 3.51 ppm were assigned to the protons of terminal methoxy group in mPEG, methylene group attached to amide bond in maleimide and methylene group attached to amide bond in mPEG. The peaks at 3.75 ppm and 7.55 ppm were assigned to the protons of mPEG backbone and methine group of maleimide, respectively. The peaks at 7.15 ppm in Figure S1E were assigned to the protons of methine group on the benzene ring of phenylboronic acid. In addition, the FT-IR spectra were collected to verify the chemical structures of the polypeptide thermogels further. As shown in Figure S1F, the signals at 1655.8 and 1547.8 cm^−1^ attributed to stretching vibration of the amide bond (νC=O) and (νC(O)-NH) of backbone further validated the chemical structures of polypeptides. The signals at 812.0 and 494.7 cm^−1^ attributed to stretching vibration and bending vibration of the carbon-hydrogen bond (νC-H and δC-H) on the benzene ring. Simultaneously, the signals at 1006.8 and 1467.8 cm^−1^ attributed to stretching vibration of carbon-sulfur bond (νC-S) and boron-oxygen bond (νB-O). This confirmed that 4-mercaptophenylboronic acid was successfully grafted to the PEG terminal of polypeptide thermogels.


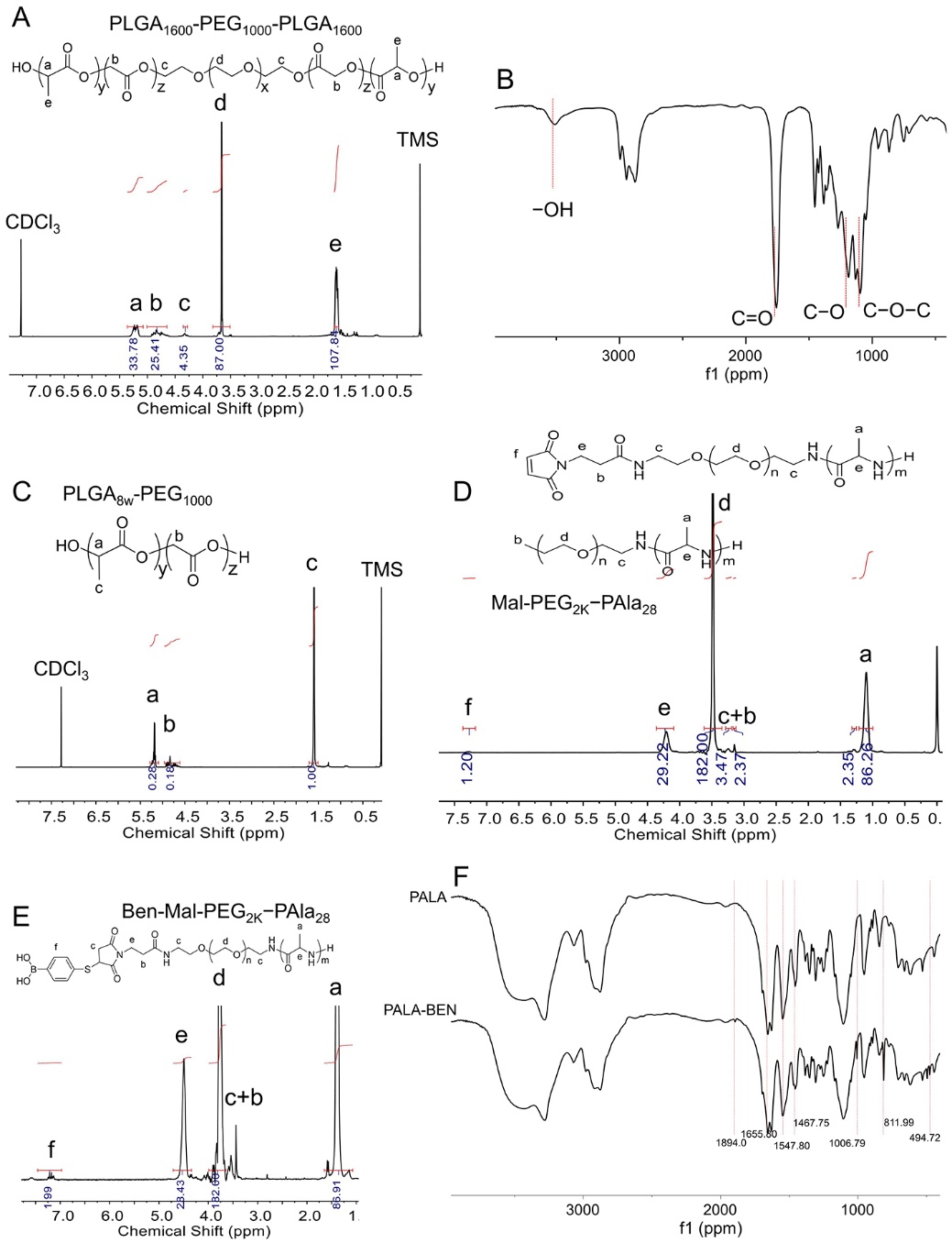


Figure S1. ^1^H NMR and FT-IR analyses. (A) ^1^H NMR and (B) FT-IR spectra of PLGA-PEG-PLGA. (C) ^1^H NMR of PLGA_8W_-PEG_1000_. ^1^H NMR of (D) Mal-PEG-b-PLAla_28_ and (E) PA-PEG-b-PLAla_28_. (F) FT-IR of Mal-PEG-b-PLAla_28_ and PA-PEG-b-PLAla_28_.
